# Sensitization of the reinforcing value of high energy density foods is associated with increased zBMI gain in adolescents

**DOI:** 10.1038/s41366-021-01007-w

**Published:** 2021-11-30

**Authors:** Jennifer L. Temple, Amanda M. Ziegler, Amanda K. Crandall, Tegan Mansouri, Lori Hatzinger, Rachel Barich, Leonard H. Epstein

**Affiliations:** 1 Department of Exercise and Nutrition Sciences, School of Public Health and Health Professions, University at Buffalo, Buffalo, NY 14214 USA; 2 Department of Community Health and Health Behavior, School of Public Health and Health Professions, University at Buffalo, Buffalo, NY 14214 USA; 3 Department of Pediatrics, Jacobs School of Medicine and Biomedical Sciences, University at Buffalo, Buffalo, NY 14214 USA

**Keywords:** Preclinical research, Risk factors

## Abstract

**Background/Objectives:**

Characterizing behavioral phenotypes that predict increased zBMI gain during adolescence could identify novel intervention targets and prevent the development of obesity. The purpose of this study was to determine if sensitization of the relative reinforcing value (RRV) of high (HED) or low energy density (LED) foods predicts adolescent weight gain trajectories. A secondary aim was to test the hypothesis that relationships between sensitization of the RRV of food and weight change are moderated by delay discounting (DD).

**Subjects/Methods:**

We conducted a prospective, longitudinal cohort study in 201 boys and girls with an average zBMI of 0.4, who began the study between the ages of 12 and 14 years and completed the study 2 years later. Participants completed five laboratory visits where the RRV of HED and LED, and DD were assessed at a baseline (visits 1, 2, and 4) and then RRV was measured again after participants consumed a portion of the same HED and LED food for 2 weeks (visits 3 and 5; order counterbalanced). Increases (>1) in the RRV from baseline to post-daily intake were categorized as “sensitization” and decreases (≤1) were categorized as “satiation.” Participants returned to the laboratory for follow-up visits at 6, 15, and 24 months to have height and weight taken and to complete additional assessments.

**Results:**

Sensitization to HED food was associated with a greater zBMI change over time (*β* = 0.0070; *p* = 0.035). There was no impact of sensitization to LED food or interaction between sensitization to HED and LED food on zBMI change and no moderation of DD on the relationship between HED sensitization and zBMI change (all *p* > 0.05).

**Conclusion:**

Our prior work showed that sensitization to HED food is cross-sectionally associated with greater zBMI. This study extends this work by demonstrating that sensitization to HED food prospectively predicts increased zBMI gain over time in adolescents without obesity. Future studies should determine if sensitization can be modified or reduced through behavioral intervention.

**Trial registration:**

Clinicaltrials.gov: NCT04027608.

## Introduction

Adolescence is a critical time period for establishing weight-related behaviors that track to adulthood [1]. This period is marked by rapid physical, psychosocial, and neurological development that favors reward responses that are often not countered by a relatively immature inhibitory control system [2–4]. In addition, increased autonomy over eating decisions may create opportunities to explore a less restrictive food environment, leading to poor eating habits that can contribute to increased weight gain that, in turn, predicts adult obesity. It is important to understand factors that contribute to these relationships in order to identify potential intervention targets.

One behavioral phenotype that is associated with obesity and obesity risk across the lifespan is the relative reinforcing value (RRV) of food, which is an empirical index of motivation to get food [5]. Higher RRV of food is associated with greater BMI and weight gain over time [5]. This relationship is moderated by delay discounting (DD), or the tendency to select smaller, more immediate rewards as opposed to larger, delayed rewards [6]. Having a high RRV of food and high DD, a combination known as reinforcement pathology, is associated with greater BMI, weight gain, and resistance to weight loss [7, 8]. Adolescence is an understudied developmental time period with respect to reinforcement pathology and may be an important time for establishing this behavioral phenotype.

While the RRV of food is conceptualized as a trait, it can be modified based on state (e.g., hunger), available alternatives (e.g., television watching), or through experience (e.g., variety or monotony of food intake) [9–14]. Repeated intake of highly liked snack foods every day for 2 weeks decreased the RRV of that food in adults without obesity, but tended to increase the RRV of food in adults with obesity [13–16]. We have conceptualized this increase in RRV after repeated intake as sensitization [17]. Sensitization has been well described in the substance use literature, and is thought to be responsible for the neurobiological changes that accompany substance abuse [18]. We have demonstrated that the sensitization of the RRV of high energy density (HED) food is associated with greater BMI and weight gain over time in adults [17] and is cross-sectionally associated with greater zBMI in adolescents [19].

Much of the prior work on RRV and on sensitization has focused on HED, highly palatable snack food, but little is known about potential protective effects of sensitization to repeated intake of healthier, low energy density (LED) foods. While sensitization is more likely to occur to high fat and/or high sugar foods, due to their ability to elicit dopamine release [20], it is possible that some people sensitize to LED foods and that this response to repeated intake of LED foods could potentially be protective against weight gain. Our prior studies in adults showed a low rate of sensitization to LED food and no relationship between RRV of LED food and weight [15]. Furthermore, a recent study published by Casperson et al. showed that repeated intake of vegetables failed to produce sensitization in the majority of participants [21]. Our previous pilot study in adolescents also showed a very low rate of sensitization to repeated LED food intake [22]. While these findings suggest that LED sensitization is rare and unrelated to weight change, these prior studies were cross-sectional, short term, and did not include interactions with HED sensitization. It is possible that sensitization to LED food could offset the impact of sensitization to HED food on zBMI change, so we explored that interaction in this study.

The purpose of this study was to examine the impact of sensitization to repeated intake of HED and LED food on zBMI change over 2 years in a large cohort of adolescents. We hypothesized that sensitization to HED food is positively associated with zBMI gain over time, that sensitization to LED food is negatively associated with zBMI gain over time, that sensitization of HED and LED would interact with one another to impact zBMI change, and that the relationship between sensitization to HED food and zBMI gain is moderated by DD.

## Methods

### General study description

This study was a 2-year, prospective observational cohort study in 201 adolescents aimed at identifying behavioral factors associated with increased or decreased zBMI change over time. The baseline data collection procedures have been described in detail previously [19]. Briefly, participants visited the laboratory five times over a 5- to 6-week period. Informed consent was obtained from parents and written assent was obtained from all adolescent participants. Participants completed a series of measures, including RRV of HED and LED food, sensitization to repeated intake of HED and LED food, DD, height and weight, food liking and wanting, hunger, thirst, and demographics. After the baseline data collection visits, participants visited the laboratory for follow-up assessments at 6, 15, and 24 months. The primary endpoint from the follow-up visits that is used in this analysis is height, weight, and zBMI. All procedures described here were approved by the Social and Behavioral Science Institutional Review Board at the University at Buffalo. This study represents the sixth replication of the sensitization paradigm [13–15, 19, 22].

### Study participants

**Fig. 1:**
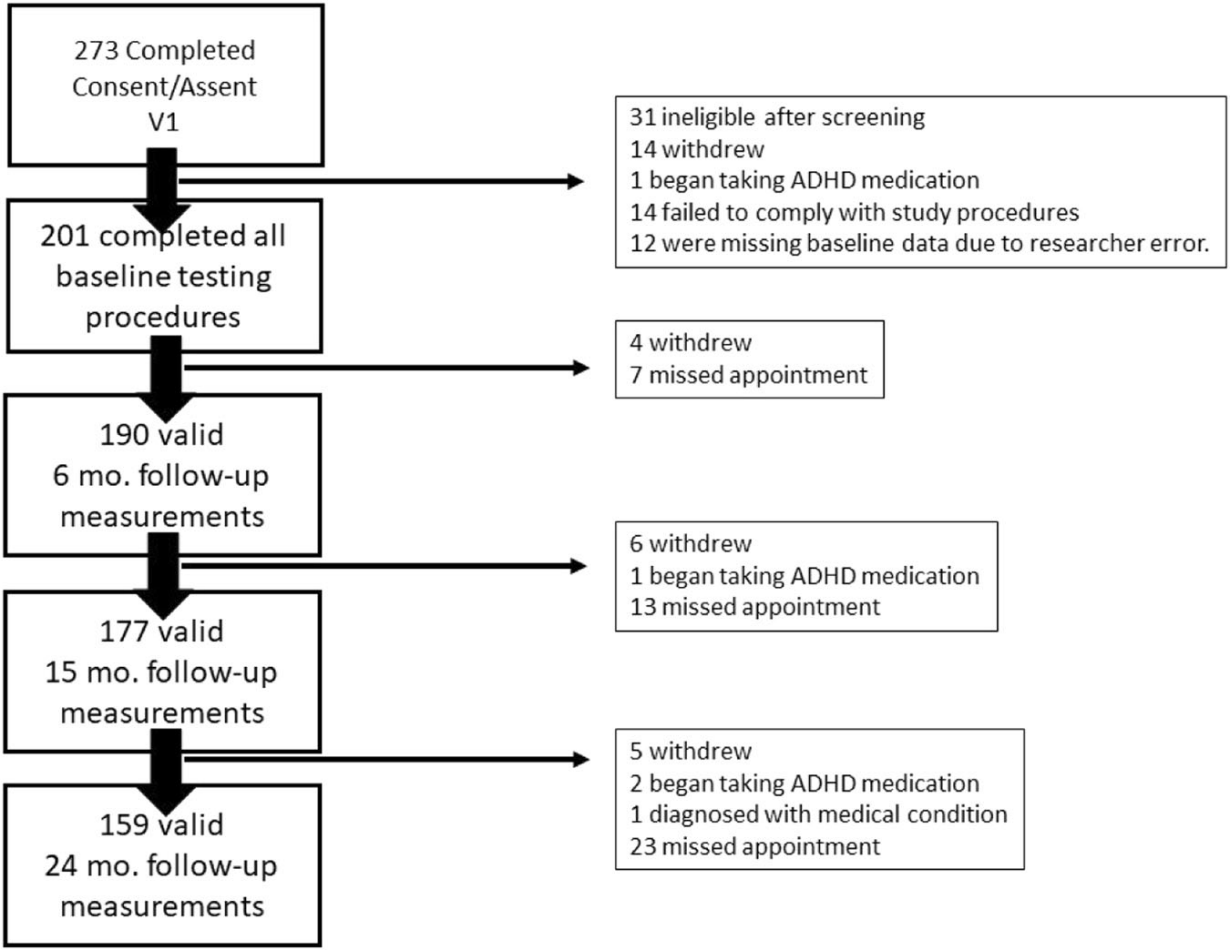
This CONSORT diagram shows participant flow through our study. The reasons why participants were not included in the study visit are listed to the right. There were participants who missed an appointment, but did not formally withdraw from the study. Those are reflected in the language “missed appointment.” In some cases, participants missed in intermediate appointment, but returned for later appointments.

Study participants were 201 adolescents aged 12–14 at baseline and 14–16 at the 24-month follow-up timepoint. Inclusion criteria were being within the age range, having a zBMI between –1.5 and 2.0 at baseline, moderate liking of at least one HED and LED food and willingness to consume those foods every day for 2 weeks, having a reasonable expectation of living in the Western New York area for the next 2 years, and able to speak and read English (both participant and consenting parent or guardian). Exclusion criteria included medication use or medical condition that could impact appetite and allergies to the study foods. We used an upper limit of zBMI of 2 to exclude participants with obesity in accordance with WHO recommendations [23, 24]. The consort diagram in Fig. 1 describes the flow of participants from consent to completion of follow-up visits.

### Anthropometrics

Height and weight were assessed in adolescents and parents/caregivers at baseline and again at the 6-, 15-, and 24-month follow-up visits. Height and weight were assessed without shoes while wearing light weight clothing using a wall-mounted, digital stadiometer (SECA; Hanover, MD) and a digital scale (SECA, Hanover, MD). Standardized zBMI values were then calculated using age- and sex-specific CDC growth charts [25].

#### Behavioral tasks

##### Relative reinforcing value task

The RRV of HED and LED food was assessed on visits 2–5 using a widely used computer task that has been validated in children, adolescents, and adults [19]. Briefly, participants were given the choice between responding on one computer for a portion of their preferred food (LED or HED) and the other computer for 2 min to engage in a highly liked sedentary activity. The LED (ED ≤ 1.0 kcal/g) food choices included: fruit cups, applesauce, and low-fat yogurt and were, on average, 62.2 g with a range of 51–67.8 g. The average energy of the LED foods was 48.3 kcals with a range from 47 to 51 kcals. The HED (>4 kcal/g) food choices included: potato and corn chips, cookies, and chocolate candy and were, on average, 16.8 g with a range from 15.8 to 18 g. The average energy of the HED foods was 90 kcals with a range from 90.0 to 90.8 kcals. The schedules of reinforcement were independent and progressive with 20 responses required for the first reinforcer and responses doubling thereafter (i.e., 20, 40, 80, 160, etc.). The highest schedule of reinforcement that was available required 5120 mouse clicks. Participants were told that they could stop working for food or activity whenever they wished and then they would be allowed to eat the food they earned and redeem the time earned for sedentary activity. The number of responses for each food on each schedule of reinforcement was plotted for each participant and an area under the curve was generated. This was the measure used to calculate sensitization.

##### Sensitization paradigm

In order to assess sensitization to repeated intake of HED and LED food, we used the paradigm developed in our laboratory and used in adults [13–16] and adolescents [19, 22] in previous studies. Participants were given 14 portions of their preferred HED or LED food to take home and consume each day for the 2 weeks in between visits 2 and 3 and visits 4 and 5. The LED food portions were approximately 200 g/160 kcals and the HED food portions were approximately 58 g/300 kcals. The order with which they received HED and LED food was randomized across participants. Participants were sent text reminders each day to consume their portion of food and they called or texted the laboratory and reported that they consumed the food and the time they consumed it. They were given no additional instructions about how or when to consume the food. All participants included in this sample reported >70% compliance on daily intake of both foods. Sensitization was calculated by subtracting the baseline area under the curve for the RRV of each food from the post-daily intake area under the curve for RRV of each food.

##### Delay discounting

DD was assessed on the first visit and on each follow-up visit at 6, 15, and 24 months using an adjusting amount task. Briefly, adolescents were asked to make a choice between getting $50 after a delay or a smaller amount of money immediately. The immediate values ranged from $0.50 to $50 and the time delays were 1 day, 2 days, 1 week, 2 weeks, 1 month and 6 months. The indifference point, the point at which the participant was equally likely to pick the immediate or delayed amount of money, was calculated for each time delay. From there, we calculated the rate of discounting (i.e., *k*) using Mazur’s hyperbolic discounting equation [26]. Indifference points across delays were checked for nonsystematic responses and scores were removed if any indifference point was greater than the preceding indifference point by 20% of the delayed amount or more [27]. Indifference points across time delays were graphed and the area under the curve was calculated. We used this measure from the baseline visit only for DD analysis.

#### Questionnaires

##### Demographics

Parents completed a demographic questionnaire at baseline and follow-up visits at 6, 15, and 24 months. This questionnaire assessed parent and child race and ethnicity, marital status, household income, education of one or both parents, and occupation.

##### Pubertal Development Questionnaire

This questionnaire asks questions about secondary sex characteristics for boys (growth of body hair, changes in voice, acne, and height) and girls (breast development, menstruation, and acne). This questionnaire was given to both the adolescent and parent, and the scores were averaged together.

##### Subjective responses

Participants were asked to rate their hunger, thirst, liking, and wanting of the foods for which they were working using a 100 mm visual analog scale anchored at 0 by “not at all” (e.g., not at all hungry or do not want to eat at all) and at 100 by "extremely" (e.g., extremely hungry). These sensations were assessed prior to consuming a preload, immediately after eating the preload, immediately prior to completing the RRV task, and immediately following completion of the RRV task. These assessments are scored as the distance in mm from 0.

##### Dutch Eating Behavior Questionnaire (DEBQ)

Participants answered questions related to their awareness of calories in food and their behaviors surrounding attempts to control or reduce the amount of food they eat. This questionnaire is often used as an assessment of dietary restraint in children and adolescents and has been shown to be related to children’s awareness of dieting [28]. Scores of the DEBQ are also related to RRV of food, sensitization, and zBMI in adolescents [19].

##### Sample size determination and analytic plan

The sample size for this study was based on our prior studies in adults [12, 15] that examined the relationship between sensitization, BMI, and weight change (effect size 0.19). We determined that, with an alpha of 0.05 and a power of 0.80, statistical significance could be achieved with a total of 180 participants.

In order to assess changes in zBMI over time and to account for missing values, we used multilevel modeling. To assess whether covariates are related to missingness, we examined baseline difference between those with complete versus incomplete data. There were no significant differences between these groups for child and parent BMI, race, sensitization, pubertal development, and food insecurity (all *p* > 0.05). We examined the scatterplots of zBMI data with each independent variable and observed that the data are linear.

To test our primary hypotheses related to zBMI change over time, appointments (level 1) were nested within individuals (level 2) and models included month of visit, sex, DEBQ score, pubertal development score, and baseline zBMI as fixed effects. Our dependent measure was zBMI at baseline and 6, 15, and 24 months. We used number of months between visits as our marker of time (i.e., 6, 15, and 24). To test hypotheses related to sensitization, we categorized people as “sensitizers” (>1) or “satiators” (≤1) depending on their change in RRV of food from baseline to post. This threshold was chosen to account for participants who increased responding from baseline to post, even by a small amount, while allowing participants who did not change responding or who decreased responding to be considered together as satiators. To test hypothesis 1, we included HED sensitization in the model and examined interactions with time. To test hypothesis 2, we included LED sensitization in the model and examined interactions with time. To test hypothesis 3, we included both HED and LED sensitization in the model, we interacted each one individually with time, and we interacted them with each other and with time. Finally, to test hypothesis 4, we added DD as a fixed effect and interacted it with HED sensitization and visit to examine moderation.

## Results

### Subject characteristics

Descriptive characteristics of our sample at baseline are stratified by sensitization status and shown in Table 1. Briefly, our sample was evenly distributed between boys and girls (52% female), largely non-Hispanic white (71%), upper-middle class (52% reported a household income >$90,000/year), and from parents who were considered well educated (68% complete college or an advanced degree). There were no differences in demographic characteristics as a function of sensitization nor were there difference in participant characteristics, such as baseline age or zBMI. The sensitizers did have a greater RRV of seated activity (*F*(1, 198) = 4.07; *p* = 0.045) at baseline compared with the satiators, but there were no other group differences in HED or LED food responses.

**Table 1:** Participant characteristics and HED and LED food measures at baseline.

	Entire sample		Satiators		Sensitizers		
	*n* = 201		*n* = 165		*n* = 36		
	*n*	%	*N*	%	*n*	%	*p*
*Sex*							
Male	96	48	77	47	20	56	
Female	105	52	88	53	16	44	0.22
*BMI percentile category*							
<85th %ile	144	72	118	72	26	72	
85th–95th %ile	40	20	32	19	8	22	
>95th %ile	17	8	15	9	2	6	0.63
*Ethnicity*							
Hispanic or Latino	14	7	11	7	3	8	
Non-Hispanic or Latino	186	93	153	93	33	92	0.58
*Race*							
American Indian/Alaska	1	1	1	1	0	0	
Native	4	2	3	2	1	3	
Asian/Pacific Islander	0	0	0	0	0	0	
Black/African American	23	11	20	9	3	8	
White or Caucasian	157	78	127	79	33	83	
Other/mixed race	16	8	14	9	2	6	0.99
*Household income*							
<$9,999	1	1	1	1	0	0	
$10,000–$49,999	33	17	25	15	8	22	
$50,000–$69,999	31	15	23	14	8	22	
$70,000–$89,999	30	15	27	16	4	11	
$90,000–$109,999	29	14	27	16	2	6	
$110,000–$139,999	36	18	30	18	6	17	0.82
>$140,000	40	20	32	20	8	22	
*Parental education*							
Completed high school	9	4	8	5	1	3	0.98
Some college/completed vocational training	28	14	23	14	5	14	
Complete college/university	83	42	68	41	16	44	
Completed graduate degree	80	40	66	40	14	39	
	Mean	SEM	Mean	SEM	Mean	SEM	*p*
Age (years)	13.3	0.06	13.2	0.06	13.4	0.15	0.41
Child zBMI (baseline)	0.40	0.07	0.38	0.08	0.48	0.14	0.58
Child zBMI (24 months)	0.44	0.07	0.40	0.08	0.62	0.17	0.26
Change in child zBMI	0.07	0.04	0.04	0.04	0.197	0.09	0.10
Parent BMI	29.5	0.46	29.2	0.51	30.8	1.1	0.17
Pubertal development	2.6	0.05	2.6	0.7	2.6	0.6	0.54
DEBQ score	4.1	0.20	4.0	0.22	4.5	0.49	0.39
*HED food measures*							
Hunger on HED visit	55.3	1.7	54.6	1.9	58.5	4.4	0.40
HED food liking	78.0	1.3	78.2	1.3	77.1	3.4	0.74
RRV of HED food	150.5	17.8	150.3	18.7	151.3	51.5	0.98
RRV of seated activity	138.5	19.9	121.8	19.1	215.3	68.3	0.07
HED sensitization	−57.2	13.0	−90.9	13.6	98.1	24.9	<0.001
*LED food measures*							
Hunger on LED visit	54.2	1.7	53.7	1.9	56.3	3.9	0.56
LED food liking	68.0	1.4	69.5	1.5	61.5	3.1	0.03
RRV of LED food	83.7	10.9	75.7	9.9	120.6	40.8	0.12
RRV of seated activity	151.9	20.1	144.3	22.3	187.0	46.5	0.42
LED sensitization	−2.5	14.0	−1.9	16.0	−5.6	27.2	0.92

### Sensitization to HED food predicts greater zBMI change over time

**Fig. 2:**
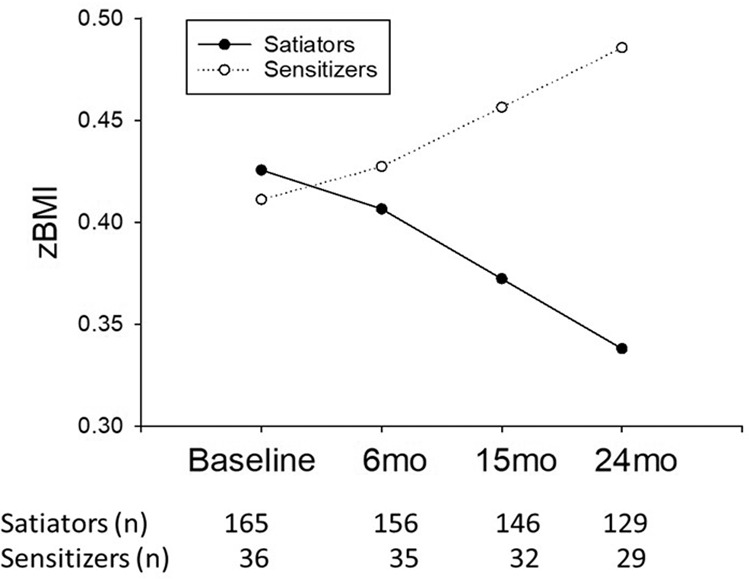
This figure shows the relationship between sensitization to repeated intake of high energy density (HED) food and zBMI change over time using the estimates from the mixed regression model. Sensitizing to repeated administration of HED food was associated with greater zBMI gain over time compared with satiators, who had a slight decrease in zBMI over time (*β* = 0.007; *p* = 0.035).

There were main effects of baseline zBMI (*β* = 0.90; *p* < 0.0001) and pubertal development (*β* = 0.014; *p* = 0.01) on zBMI. There was a significant interaction between time and HED sensitization category on zBMI (*β* = 0.0074; *p* = 0.033; Fig. 2 and Table 2). No other main effects or interactions were observed.

**Table 2:** Mixed regressions models examining zBMI in UB-SNAK participants.

	*β*	SE	*T*	*F*	*p*
*Model 1*					
Visit	−0.0038	0.0022	−1.77	3.12	0.077
DEBQ	0.0018	0.0043	0.418	0.175	0.676
Baseline zBMI	0.9058	0.0186	48.77	2378.68	<0.0001
Sex	−0.0214	0.0487	−0.439	0.193	0.661
Pubertal development	0.0113	0.0055	2.07	4.296	0.039
HED sensitization	−0.0214	0.0685	−0.312	0.097	0.755
Sex × HED sensitization	0.0111	0.0863	0.129	0.017	0.897
Visit × HED sensitization	0.0070	0.0033	2.113	4.464	0.035
*Model 2*					
LED sensitization	−0.0824	0.0602	−1.367	1.869	0.173
Sex × LED sensitization	0.1743	0.0741	2.354	5.540	0.019
Visit × LED sensitization	0.0013	0.0029	0.469	0.220	0.639
*Model 3*					
HED sensitization	−0.0367	0.0683	−0.537	0.288	0.592
LED sensitization	−0.0052	0.0567	−0.091	0.008	0.928
HED × LED sensitization	0.0609	0.1318	0.462	0.213	0.644
Visit × HED × LED sensitization	0.0068	0.0079	0.850	0.723	0.395
*Model 4*					
Delay discounting (DD)	−0.0007	0.0038	−0.181	0.033	0.857
HED sensitization × DD	−0.1263	0.1563	−0.808	0.184	0.668
DD × Visit	−0.0001	0.0003	−0.509	0.259	0.611
Sex × DD	0.0008	0.0076	0.110	0.012	0.913
Sex × HED sensitization × DD	0.0074	0.1539	0.481	0.232	0.630
Sex × DD × Visit	−0.0002	0.0005	−0.400	0.160	0.690
Visit × HED sensitization	0.0102	0.0061	1.669	2.785	0.096
HED sensitization × DD × Visit	0.0003	0.0008	0.405	0.164	0.685

All models include baseline zBMI, DEBQ score, sex, and pubertal development score as fixed effects and visit as a repeated measure. Model 1 adds sensitization to HED food and interactions between HED sensitization and visit and HED sensitization, visit, and sex. Model 2 adds sensitization to LED food and interaction between LED sensitization and visit and LED sensitization, sex, and visit. Model 3 adds both HED and LED sensitization, interactions with each individually and visit, interactions between both LED and HED sensitization and visit, and interactions among LED and HED sensitization, visit, and sex. Model 4 adds DD, interactions between HED sensitization and DD, sex and DD, DD, HED sensitization, and visit, and DD, HED sensitization, visit, and sex.

### Sensitization to LED food not related to zBMI change over time

When we examined sensitization to LED food we found no main effect of LED sensitization (*β* = 0.006; *p* = 0.91) and no interaction between LED sensitization and time (*β* = –0.002; *p* = 0.58).

### There is no interaction between sensitization to HED and LED food on zBMI change over time

When we included both HED and LED sensitization in the model, there were no main effects of HED (*β* = −0.04; *p* = 0.59) or LED (*β* = −0.005; *p* = 0.93) sensitization on zBMI and no interactions between LED and HED sensitization (*β* = 0.061; *p* = 0.64) and no interactions between HED sensitization, LED sensitization, and time (*β* = 0.007; *p* = 0.39).

### Delay discounting does not moderate the interaction between sensitization to HED food and zBMI change over time

When we include DD in the model from hypothesis 1, there are no main effects of DD on zBMI (*β* = −0.0007; *p* = 0.86) or zBMI change over time (*β* = −0.0001; *p* = 0.61). There were also no interactions between DD and HED sensitization (*β* = 0.0036; *p* = 0.67) or among HED sensitization, DD, and time (*β* = 0.0003; *p* = 0.69).

## Discussion

Incentive sensitization theory has been well described in the context of substance use, but its application to eating behavior has been limited. Our prior work in adults, informed by this theory, demonstrated that sensitization of the RRV of HED food after repeated intake is cross-sectionally and prospectively associated with BMI and weight gain. The purpose of this longitudinal cohort study was to extend this work into adolescents without obesity and to examine the relationship between sensitization to HED and LED food and the influence of DD on zBMI change over time. Our main finding was that sensitization to repeated HED food predicts greater zBMI gain over 2 years. There were no interactions with sensitization to LED food or with DD. These results show that individuals who sensitize to repeated intake of HED food have accelerated zBMI gain relative to peers who do not demonstrate this phenotype. Sensitization of the RRV of food may be an important and novel intervention target for the prevention of increased zBMI gain in children and adolescents.

Our prior worked showed that adults with obesity were more likely to sensitize than those with healthy weight [13, 15], that sensitization required larger portions of HED food [13], that repeated intake of LED food rarely resulted in sensitization [15], and that adults who sensitized gained more weight than those who satiated [17]. Sensitization to repeated intake of HED food is consistent with the literature showing that highly palatable foods, in particular those containing high sugar, activate the neural reward systems more strongly than other types of food [29]. In our baseline data analysis from the current cohort, we found that sensitization to HED was associated with baseline zBMI [19], but that only a small percentage of our sample of over 200 adolescents without obesity sensitized to HED food (15%), compared with over 30% of adults [13]. The findings presented here demonstrate two things. First, that sensitization is more prevalent in adults than in adolescents, suggesting that it develops later in life, perhaps after increased weight accumulation. Second, that the relationships between sensitization to repeated intake of HED food and weight and weight gain are consistent and reproducible in adolescents and adults.

Much of the prior work on RRV of food and on sensitization has focused on HED foods. The few studies that have examined the RRV of LED foods have had mixed results. Our previous studies in adults and adolescents showed that LED foods did not tend to result in sensitization [15, 22]. A more recent paper that used a more intensive sensitization protocol also found that repeated intake of vegetables did not result in sensitization in adults [21]. In the current study, we examined baseline RRV of LED food as well as LED sensitization. We hypothesized that LED sensitization would be protective against increased zBMI gain in our adolescents, but found no relationship between LED sensitization and zBMI change over time. This could be because LED sensitization can occur along with HED sensitization and greater intake of HED food, so LED sensitization alone does not necessarily mean that adolescents are eating healthier or consuming less energy. In order to examine these potential interactions, we analyzed the interactions between HED and LED sensitization and also found no significant interactions or relationships with zBMI change. This suggests that HED sensitization is the primary risk factor for increased zBMI gain and that this occurs in individuals who do and do not sensitize to repeated intake of LED food.

Another factor that could influence zBMI gain is DD. Prior work has shown that higher DD and reinforcement pathology are associated with greater BMI and greater weight gain over time [30, 31]. We tested the hypothesis that higher DD is associated with greater zBMI gain over time and that DD moderates the relationship between HED sensitization and zBMI gain. Contrary to our hypotheses, we found no main effects or interactions of DD on zBMI change. There are a number of explanations for this finding. DD is highly variable in adolescents, which may have made it difficult to observe these interactions. Another factor to consider is that given the maturation of the reward systems and the relative immaturity of inhibitory control, it is likely that, during adolescence, the regulation of eating and body weight is largely driven by motivational systems and the role of inhibitory control systems is relatively weak. DD and sensitization may also overlap, making it difficult to observe an independent contribution of DD above and beyond what is observed with sensitization. It is also important to note that when DD was included in the model, the interaction between sensitization and time on zBMI was reduced to a statistical trend. This is likely due to a reduction in power in the more complex model, as our original power calculation did not account for the addition of DD to the model.

This study has some significant strengths. First, it was a large cohort study with multiple in-person measurements over 2 years. Second, we conducted rigorous laboratory-based measures of anthropometrics, RRV of food, and DD. Third, we assessed adolescents across the bulk of pubertal development. Finally, we examined the RRV of both LED and HED food. It is also important to consider the study weaknesses. First, the sample was largely white and upper-middle class. This meant that we were underpowered to examine interactions with income, race, education, or food insecurity. Second, using a beginning age of 12 years meant that girls entered the study further along in pubertal development than boys, on average. This may have created different starting points in terms of growth velocity. Third, as expected, our sample size was highly imbalanced between sensitizers and satiators, which limited our statistical power. Finally, our follow-up timing was impacted by the COVID-19 pandemic. Our laboratory was shut down from March 15, 2020 to July 1, 2020. At this point, we were finishing the 24-month assessments on the second half of our cohort. We stayed in contact with our families and communicated about our safety precautions once we reopened. We were able to reschedule almost all families, with a loss of only three families who disclosed discomfort with in-person assessments related to COVID-19. While we retained 80% of our original, complete cohort, our data could have been strengthened by greater retention numbers.

In sum, this study demonstrates that sensitization of the RRV of HED food after repeated intake is a novel behavioral risk factor for increased weight gain. This replicates and extends our earlier findings from adults. Sensitization of the RRV of LED food does not reduce risk of weight gain and DD does not increase it. Furthermore, neither of these factors moderates the primary relationship between the sensitization of HED food and weight gain. The stability of this phenotype and the reproducibility of its relationship with weight and weight gain suggest that sensitization may be a promising intervention target for prevention of weight gain. Future studies should investigate ways to reduce sensitization to HED food. This study shows that sensitization of LED food and DD are not good candidates for intervention targets, as they had little impact on the relationships. A focus on increasing the RRV of non-food alternatives may reduce sensitization and greater weight gain over time. This study is an important first step in the process of identifying novel behavioral targets for weight gain prevention.

## Data Availability

Data described in the manuscript, code book, and analytic code will be made available upon request pending review by corresponding author.
